# The WELLMESS Project (Move Well, Eat Well, Sleep Well, Stress Well)

**DOI:** 10.7759/cureus.68194

**Published:** 2024-08-30

**Authors:** Deeksha R Machireddy, Claudia G Reid, Joshua C Hollingsworth, David Redden

**Affiliations:** 1 College of Medicine, Edward Via College of Osteopathic Medicine (VCOM-Auburn), Auburn, USA; 2 Pharmacology, Edward Via College of Osteopathic Medicine (VCOM-Auburn), Auburn, USA; 3 Research and Biostatistics, Edward Via College of Osteopathic Medicine (VCOM-Auburn), Auburn, USA

**Keywords:** stress management, health behaviors, lifestyle changes, wellness, medical education

## Abstract

Introduction

Medical education is a rigorous and demanding journey that requires intellectual ability and emotional resilience. Burnout among medical students is a growing concern as it affects individuals' well-being and can have long-term implications on the quality of patient care. Recognizing the protective effects of healthy lifestyle factors such as sleep, physical activity, eating patterns, and screen time, various institutions have implemented wellness programs to promote students' mental and physical well-being.

Objectives

The main objective of this study was to assess for variations and associations between first- and second-year osteopathic medical students’ physical activity, eating patterns, sleep, perceived stress, and phone screen use across 10 different time points throughout the school year. The secondary objective was to assess the impact of month-long health behavior challenges among participants in the five areas studied.

Methods

A prospective cohort study was performed regarding first- and second-year medical students’ physical activity, eating patterns, sleep, perceived stress, and phone screen use, as respectively assessed by the Physical Activity Vital Sign and smartphone step count data; Starting the Conversation diet assessment; Single Item Sleep Quality Scale and a portion of the Pittsburgh Sleep Quality Index; Perceived Stress Scale; and smartphone screen time use data. The assessments, built-in Qualtrics (Qualtrics International Inc., Provo, USA), were sent via email and class-wide GroupMe text message at 10-time points, capturing the beginning, middle, and end of curricular blocks and seasonal breaks. Participants could win one of 20 $25 Amazon gift cards each time they completed the assessment. Behavior challenges were implemented at six of the 10-time points and focused on facilitating health behavior change among participants.

Results

The response rate for the 10 assessments was low, ranging from 7% to 13%. The means (±SD) for each outcome of interest combined across the 10 time points were as follows: physical activity = 112 (±100) minutes/week; step count = 4,627 (±3179) steps/day; water intake = 5.6 (±4.0) cups/day; diet score = 6.3 (±2.1) where 0 = most healthful and 16 = least healthful; perceived stress = 6.6 (±5.8) where 0 = lowest and 40 = highest; sleep = 6.7 (±1.0) hours/night; sleep quality = 6.3 (±1.8) where 0 = terrible and 10 = excellent; and phone screen time = 328.5 (±147) minutes/day. Statistical analysis indicated variation (p<0.05) in physical activity minutes, steps per day, water intake, diet score, and phone screen time minutes over time. Given low participation, only 6 of the 10 behavior challenges were implemented, in which the participation rate ranged from 36% to 65% of assessment participants.

Conclusion

Results indicate room for improvement regarding lifestyle factors among medical students’ physical activity, eating patterns, sleep, perceived stress, and phone screen time use. A primary limitation of this study is the low participation rate. Building on these efforts, students are developing and delivering monthly wellness workshops, including a research component.

## Introduction

Medical education is a rigorous and demanding journey that requires intellectual ability and emotional resilience. Throughout this process, medical students often encounter significant stressors that can lead to burnout. Burnout among medical students is a growing concern as it affects the well-being of individuals and can have long-term implications for the quality of patient care [1]. Recognizing the detrimental effects of burnout on medical students, various institutions have implemented wellness programs to promote mental and physical well-being [2]. These programs often include mindfulness training, counseling services, and initiatives to foster a supportive learning environment. While the concept of wellness programs is gaining traction, the effectiveness of these interventions in mitigating burnout and improving overall student well-being remains an area of active research.

Wellness programs typically target the holistic well-being of medical students, addressing physical, mental, and spiritual dimensions. Known benefits of exercising regularly [3], eating healthy [4], getting enough quality sleep [5], and managing stress include a lowered risk of chronic medical conditions, decreased risk of all-cause mortality, and improved cognitive functioning. According to the American Heart Association (AHA), adults should perform at least 150 minutes of moderate-intensity aerobic activity per week or 75 minutes of vigorous aerobic activity per week [6]. Additionally, the AHA recommends adding moderate- to high-intensity muscle strength resistance training at least two days per week [6]. The American Academy of Sleep Medicine recommends at least seven hours per night to promote optimal health in adults [7]. With these benefits and recommendations in mind, we decided to investigate medical student's practices related to healthy behaviors of physical activity, eating, sleeping, and managing stress.

Literature has pointed out that medical students have exhibited increased rates of burnout and depression by the time of graduation. COVID-19, the stress of licensure exam preparation, and the increasing cost of medical school all add to the mix of mental illness, substance abuse, and burnout in the medical student population [8]. Incoming medical students have been shown to enter medical school with better mental health indicators than peers in the same age class in other professions. However, there is a decrease in these indicators by the time of graduation. The medical school culture and personal factors could contribute to these findings [9]. Some categories that have been cited to be of particular stress in medical students include talking with psychiatric patients, the effects of medical school on personal life, presenting cases to attending physicians, and dealing with death and suffering in the clinical setting [10]. High levels of stress have been shown to support a causal relationship to cardiovascular diseases attributed to unfavorable changes in health behaviors and a moderately increased risk of incident coronary heart disease [11].

Additionally, increased screen time has been linked to adverse health behaviors in medical students, including delayed bedtime, shorter sleep duration, and poorer sleep quality. The unique challenges of academic stress place medical students at a heightened risk for these harmful health behaviors. Research conducted at the Technical University of Dresden revealed that medical students who reported more screen time for leisure went to bed significantly later, and students who spent more screen time studying tended to sleep for shorter periods. The results from this study indicate a need for interventions related to education and promotion of healthy sleep behaviors in this population [12]. A study in Saudi Arabia by Almojali et al. demonstrated a correlation between high-stress levels, low grade point average (GPA), and poor sleep quality among medical students [13]. The pressure of managing academic workload often leads medical students to sacrifice sleep, exacerbating the impact of stress on their well-being [13].

Additionally, the *Journal of Clinical Sleep Medicine *emphasizes the vital role of healthy sleep in various aspects of well-being [14]. Beyond its influence on cognitive functioning, mood, and mental health, sufficient and quality sleep is integral to cardiovascular, cerebrovascular, and metabolic health. Moreover, maintaining an adequate sleep regimen is associated with a reduced risk of accidents and injuries related to sleepiness and fatigue. Chronic insufficient sleep is related to an increased risk of mortality and can contribute to the development of cardiovascular disease, diabetes, obesity, and cancer [14].

Physicians who incorporate regular wellness practices into their routines have reported significantly reduced burnout and stress levels. This positive outcome is associated with increased compassion for patients and a heightened ability to provide effective counseling. Notably, research has indicated an inverse correlation between domains of burnout, particularly emotional exhaustion, and the expression of empathy among physicians [15,16]. Similar trends are observed in medical students who actively engage in self-care by adopting healthy habits. It has been highlighted that medical students practicing self-care report a decrease in stress levels and an overall improvement in their quality of life [17]. In a study conducted by Brazeau et al., surveys completed by fourth-year medical students showed that higher medical student burnout was associated with lower empathy and professionalism. There are limited studies investigating the relationships between these findings [16].

With many established wellness programs in U.S. and Canadian medical schools, most mainly focus on prevention and reaction programming without considering the structural characteristics of the programs. One such program is the Vanderbilt Medical Student (VMS) Wellness Program, which combines proactive and reactive approaches [17]. The program included mentoring/advising programs, student leadership for advocacy, and personal growth activities focused on mind, body, social, and community well-being. The program has not shown any objective improvements in student health, but anecdotal evidence shows that the programs positively influence students [17]. The effectiveness of these wellness programs should be evaluated in terms of student well-being to help with financial allocation and program development [9]. Associations and longitudinal trends regarding physical activity, eating habits, quality of sleep, perceived stress, and phone screen use time in medical students in the U.S. have not been investigated. With these points of interest in mind, this study has the potential to provide insight into time points in which medical students may benefit from additional support and available interventions.

## Materials and methods

This study was evaluated and approved by the institution’s Institutional Review Board. An institution-based application was completed and approved on 6/15/2022 before the conduction and implementation of this study and was given the approval number 2022-037. This study was extramurally funded by the American Association of Colleges of Osteopathic Medicine (AACOM). The items included in the funded budget were 200 $25 Amazon gift cards, with the entire budget totaling $5,000.00. The return of the questionnaire implied consent. An information letter was included at the beginning of each survey (assessment), leading to the implied consent. As an incentive, students could win one of 20 $25 Amazon gift cards for each assessment completed. The probability of winning a gift card was (20/320) 6.25% or better for each assessment completed (by J.H.).​

The subject population included all (approximately 320) first- and second-year osteopathic medical students during the 2022-2023 curricular year. Recruitment of subjects was completed with in-person class announcements and class GroupMe advertisements by co-investigators of the study (C.R., D.M.). Students were introduced to the survey and the incentive through recruitment and could choose whether to participate.

A prospective cohort study was performed regarding the population’s self-reported data on exercise and physical activity, eating patterns, sleep, perceived stress, and phone screen use at ten different time points during the school year by a created Qualtrics electronic survey (Qualtrics International Inc., Provo, USA). The Exercise Vital Sign [18] assessed average weekly aerobic physical activity. Average weekly strength training days were added to the survey to follow the American College of Sports Medicine’s Exercise is Medicine initiative [19] recommendations. Step counts were measured based on individual smartphone-recorded step counts reported by the participant [20]. Eating patterns were assessed with the Starting the Conversation self-assessment [21,22]. Sleep duration, efficacy, and latency were evaluated with the first five items from the Pittsburgh Sleep Quality Index [23], and the Single Sleep Quality Scale [24] was additionally used to assess self-reported sleep quality. Perceived stress was evaluated based on the Perceived Stress Scale [25]. Individual smartphone data was used for participants to report their average daily screen time use. Finally, students’ ages, genders, years in medical school, and email addresses were collected for data use and follow-up. The assessments were completed using the Qualtrics online survey software tool during the beginning, middle, and end of each curricular block and school seasonal breaks (J.H.).

Assessments were sent out via email and class-wide GroupMe text message at 10-time points, capturing the beginning (July, January), middle (August, November, February, and May), and end (September, December, March, and June) of curricular blocks (C.R., D.R.). Table 1 provides the month of each assessment paired with its curricular block. The block schedule at this institution consists of four 12-week blocks per year. Blocks 1-4 are included in year one of medical school and blocks 5-8 are included in year two.

**Table 1:** This table illustrates the 10 months during which the survey was administered, along with the corresponding blocks.

Month of Assessment	Block Timing
July	Beginning of blocks 1 and 5
August	Middle of blocks 1 and 5
September	End of blocks 1 and 5
November	Middle of blocks 2 and 6
December	End of blocks 2 and 6
January	Beginning of blocks 3 and 7
February	Middle of blocks 3 and 7
March	End of blocks 3 and 7
May	Middle of blocks 4 and 8
June	End of blocks 4 and 8

Participation was voluntary, and students who completed the survey at each time point were then entered into the randomized drawing for a $25 Amazon gift card. After completing each survey, students were emailed a summary of their results and allowed to join the monthly health behavior challenge. Behavior challenges were implemented with each time-point survey and focused on tools to improve individual health-related behaviors (J.H.).

Statistical analysis of the survey results was completed. To account for repeated measurements on the same subject and avoid case-wise deletion for missing values, generalized estimating equations were used to test for differences between observation periods. To limit the number of comparisons, post-hoc comparisons were conducted comparing the observation period with the smallest observed mean to the remaining nine periods. A type I error rate of 0.05 was used. Analyses were conducted using SAS9.4 (SAS Institute Inc., Cary, USA) (D.R.).

## Results

The response rate for the 10 assessments was low, ranging from 7% to 13% (N = 25-48). With this low participation rate, only 6 of the 10 post-survey behavior challenges were implemented. The participation rate for the behavior challenges ranged from 36% to 65% of assessment participants.

The mean (± SD) for each of the five areas of interest across the 10-time points was calculated and assessed. The mean physical activity minutes per week equaled 112 (± 100) minutes/per week. The mean step count per day was 4,627 (±3,179) steps/day. Water intake measured in cups per day reported by participants gave a mean of 5.6 (±4.0) cups per day. The mean diet score was 6.3 (±2.1), where 0 was equal to most healthful and 16 was equal to least healthful based on the Starting the Conversation diet assessment utilized [19]. The perceived stress score equaled a mean score of 6.6 (±5.8), where 0 was the lowest score (low stress), and 40 was the highest (high stress) based on the Perceived Stress Scale [23]. The average hours of sleep per night was 6.7 (±1.0), with a mean sleep quality score of 6.3 (±1.8). The sleep quality score was measured based on a portion of the Pittsburgh Sleep Quality Index [21] and the Single Item Sleep Quality Scale [22], where a score of 0 was equal to terrible sleep quality and 10 was equal to excellent sleep quality. The mean phone screen time measured in minutes per day was 328.5 (± 147) minutes/day.

**Figure 1:**
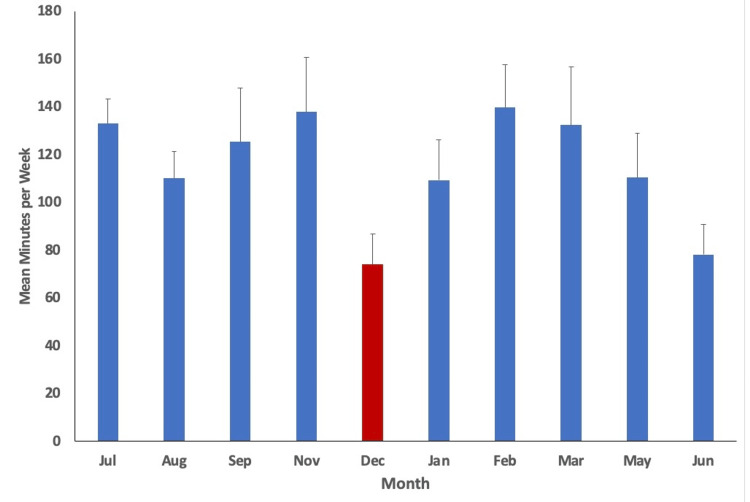
Mean physical activity minutes per week for each month measured. The smallest mean measured is indicated in red (December).

**Figure 2:**
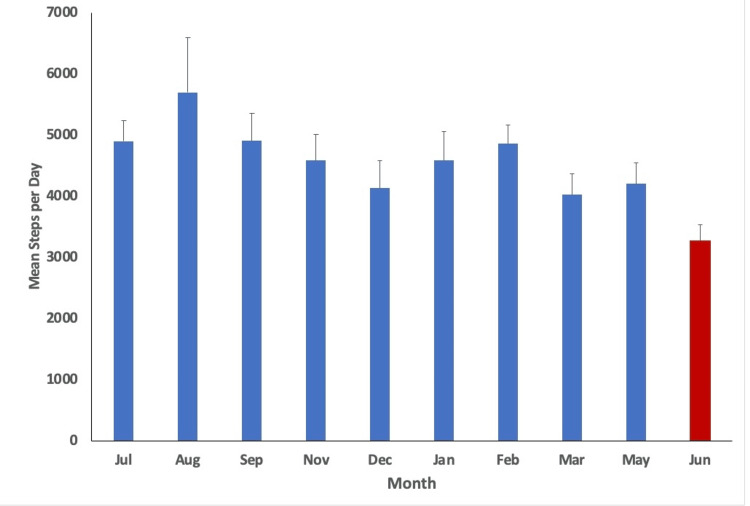
Mean steps per day for each month measured. The smallest mean measured is indicated in red (June).

**Figure 3:**
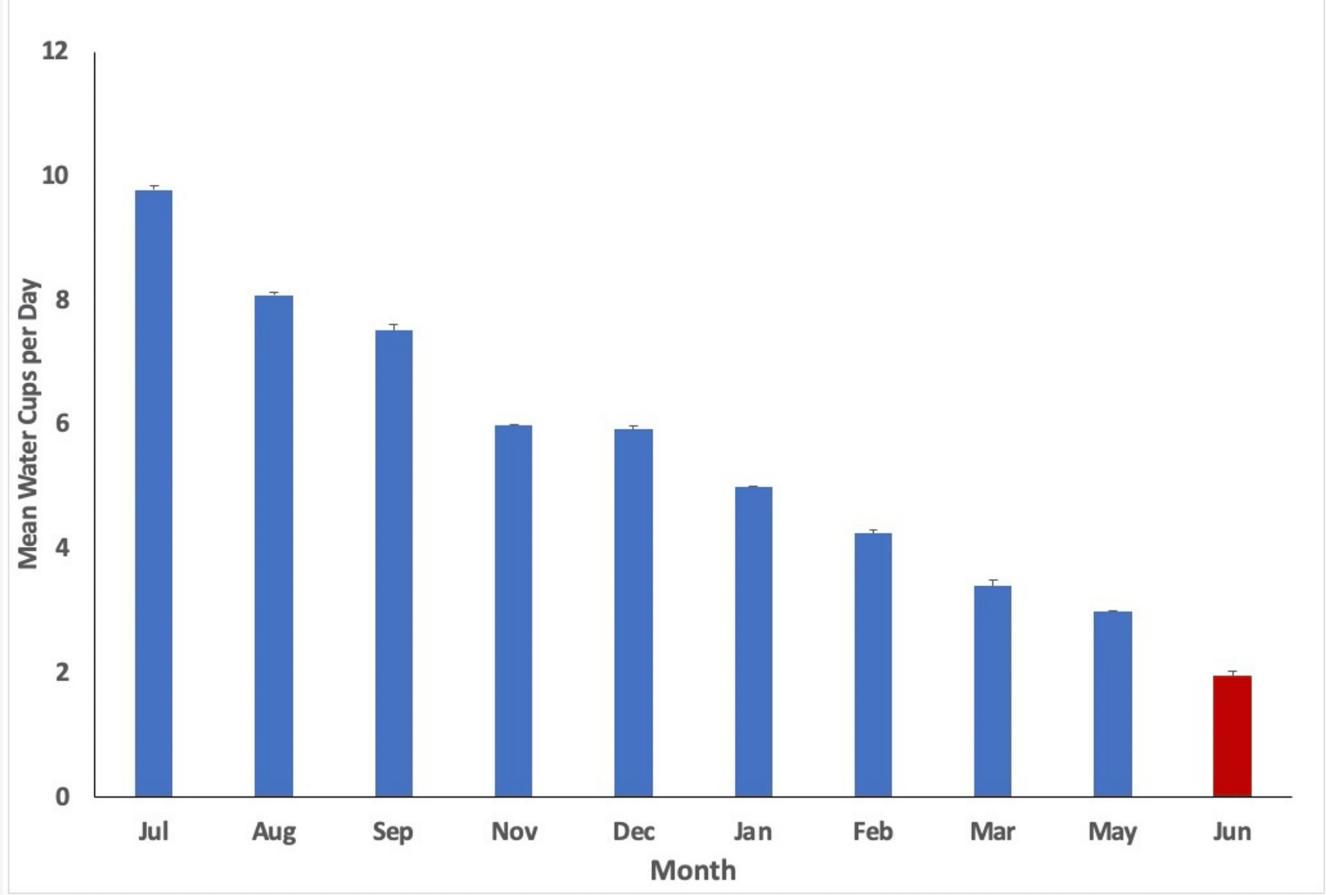
Mean cups of water per day for each month measured. The smallest mean measured is indicated in red (June).

**Figure 4:**
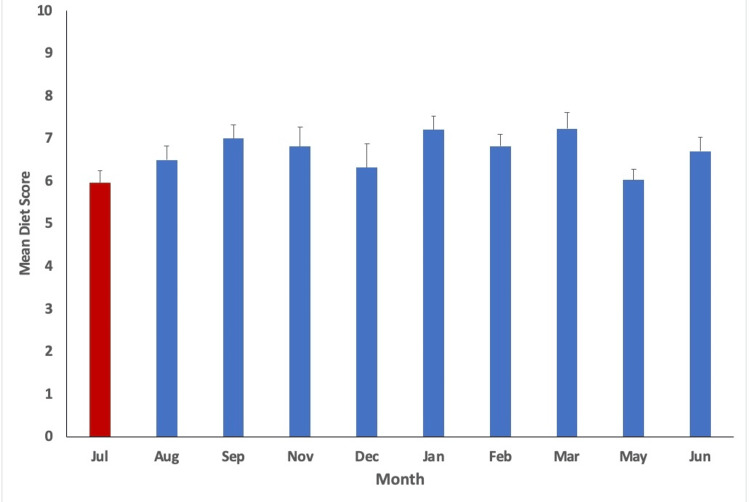
Mean diet score for each month measured. The smallest mean measured is indicated in red (July).

**Figure 5:**
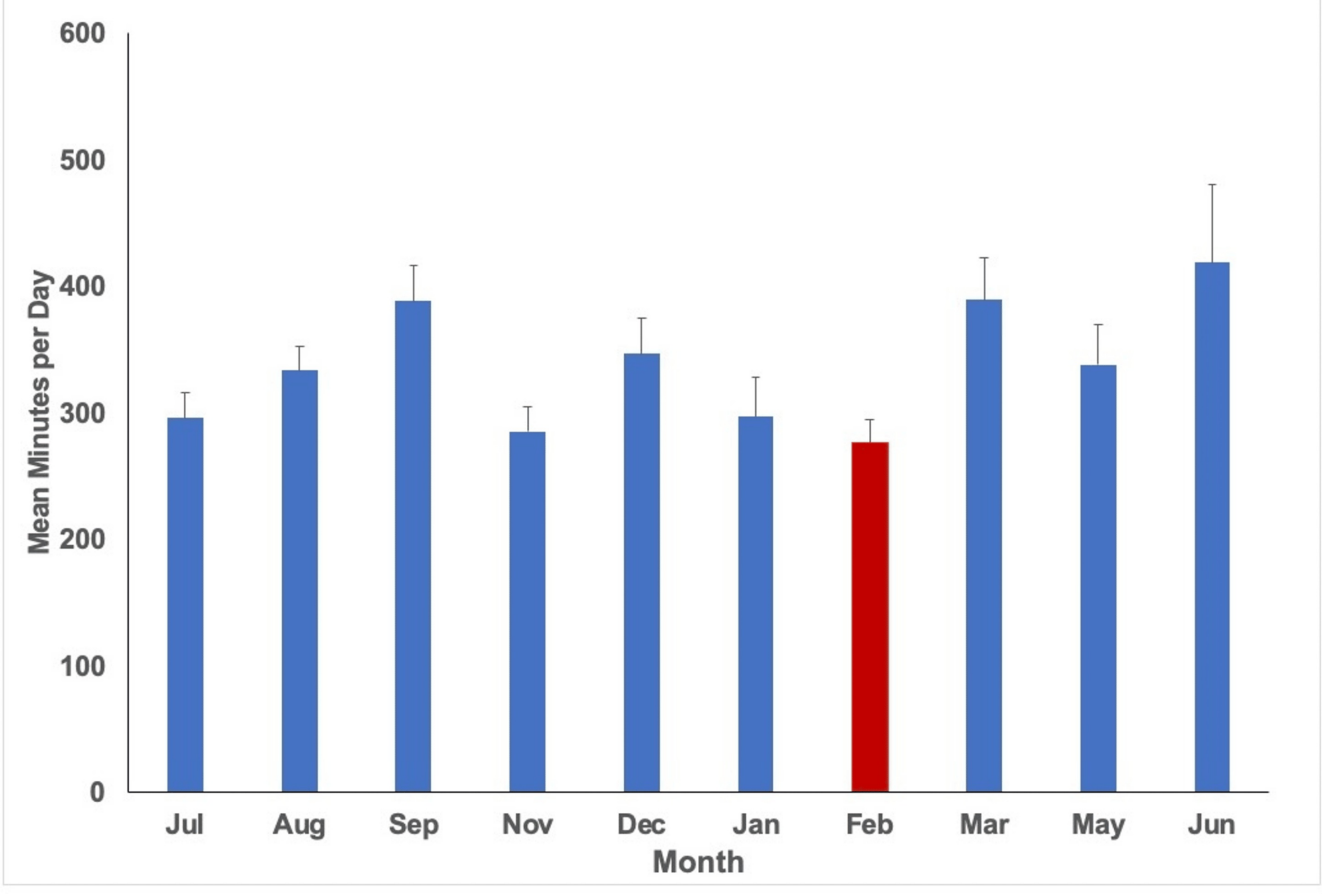
Mean phone screen minutes per day for each month measured. The smallest mean measured is indicated in red (February).

With a p-value of less than 0.05 utilized (p<0.05), statistical analysis indicated variation in physical activity (minutes/week), step count (steps/day), water intake (cups of water/day), diet score (0-16), and phone screen time (minutes/day) over time. The mean values per month measured alongside their respective standard error are plotted in Figures 1-5, with the red bar indicating the smallest mean observed. The smallest means with their respective measurements were found as follows: physical activity 74.1 (± 12.7) minutes/week, step count 3,272.4 (±255.1) steps/day, water intake 1.9 (±0.1) cups/day, diet score 6.0 (±0.3), and phone screen time 276.4 (±17.9) minutes/day.

## Discussion

The results of this study provide valuable insights into the lifestyle behaviors and perceived stress levels of first- and second-year osteopathic medical students. Understanding these factors is a crucial step for developing effective interventions to promote the well-being of medical students and ultimately improve patient care.

Based on the study results, steps per day, water cups per day, and weekly physical activity minutes showed the lowest outcomes at the conclusion of curricular blocks. This may be attributed to increased stress and time commitments at the end of blocks. Burnout, or physical and mental exhaustion [26], often manifests when professional stress conflicts with personal ideals or expectations [8]. With final examinations, deadlines and due dates, and final class grades being published, burnout is a crucial concern to remember near the end of curricular blocks due to the physical and mental strain each item can cause. Klein and McCarthy identify dissatisfaction with the learning environment and lack of faculty support to be the two main areas of dissatisfaction and burnout in preclinical medical students [8]. With this in mind, it could be proposed that as students feel burnout and dissatisfaction after working hard for an entire block, they may feel less motivated to maintain physical wellness practices.

Steps per day and water cups per day recorded their lowest measurements in June, coinciding with the conclusion of blocks 4 and 8, which mark the end of the school year according to the institution’s academic calendar. This period may also be associated with burnout, potentially explaining the decline in these health-related behaviors. Pass/Fail grading systems have been implemented by some institutions to see if the change affected wellness-related metrics and burnout among medical students. Rhoe et al. determined that first-year medical students at Mayo Medical School utilizing a Pass/Fail grading system reported less stress, better overall mood, and greater cohesion than their graded peers [27]. This could be an area of continued research and consideration for medical schools considering avenues to increase student wellness.

Phone screen time demonstrated a pattern of being lowest in the middle of blocks 3 and 7 while reaching its peak during months corresponding to the end of academic blocks. Several factors could have contributed to these findings, including increased phone screen time usage for studying, elevated burnout, and academic workload. Prolonged screen time has been associated with various adverse health outcomes, including disrupted sleep patterns, decreased physical activity, and impaired social interactions. Implementing strategies to promote mindful technology use, such as setting boundaries and engaging in digital detoxes, is crucial for mitigating the adverse effects of excessive screen time on overall well-being.

Additionally, the diet score was lowest, or most healthful, in July, aligning with the commencement of blocks 1 and 5. This suggests that students might be more health conscious and mindful of their dietary habits at the beginning of new academic years, aiming for a healthier start. Again, at the beginning of blocks, students most likely have not yet reached the point of physical and mental exhaustion due to coming from a preceding school curriculum break, so the increased healthful behavior can most likely be expected before burnout and observed associated decrease in wellness behaviors sets in. Poor dietary choices among medical students can lead to nutritional deficiencies, fatigue, and impaired concentration, all of which can adversely affect academic performance and clinical decision-making.

Perceived stress levels among participants were moderate, with variations observed over time. Medical education is inherently stressful, characterized by academic pressures, long hours of study, and exposure to emotionally challenging clinical experiences. Chronic stress not only compromises mental health but also weakens the immune system and increases susceptibility to burnout and compassion fatigue. Interventions aimed at promoting stress management skills, such as mindfulness-based practices and cognitive-behavioral techniques, are essential for equipping medical students with the resilience necessary to navigate their training demands.

Implementing behavior challenges as part of this study represents a promising approach to promoting health behavior change among medical students. While participation rates varied across challenges, the positive response from participants underscores the potential effectiveness of tailored interventions in fostering lifestyle modifications. Future research should explore innovative strategies for increasing engagement and sustaining behavior change over time.

Additional efforts are underway to enhance student involvement in wellness initiatives by developing monthly wellness workshops at the institution. These workshops, which students would lead, will aim to improve well-being and include a research component to strengthen future data collection and analysis. While planning these workshops, student’s perceived stress levels associated with time commitment and additional workload will be considered to avoid adding to the current workload and burden. Wellness initiatives have been shown to be more effective when they reduce student burden instead of adding additional requirements [28]. When considering wellness initiatives, it is worth considering how the initiative could reduce student burden by not giving them extra work but alleviating some of the current workload. Medical student’s schedules do not leave much extra time to engage in additional wellness programming. Lonka et al. identified that a collaborative approach to learning increased satisfaction and decreased the perceived workload [29]. Adding self-care workshops into the curriculum has been shown to decrease the depersonalization component of burnout [28]. These observations lead to the need for a multifactorial approach to create a wellness program that helps decrease burnout in students while promoting wellness-related behaviors and not adding additional stress due to scheduling and additional commitments.

Limitations

Despite the valuable insights gained from this study, several limitations must be acknowledged. The low response rate for assessments raises concerns regarding the generalizability of the findings and potential selection bias. Future studies should employ strategies to enhance participation, such as incentivizing completion and utilizing multiple communication channels for outreach. Additionally, the reliance on self-reported data introduces the possibility of recall bias and social desirability bias, which may influence the accuracy of responses.

## Conclusions

In conclusion, this study highlights the importance of addressing lifestyle behaviors and perceived stress levels among medical students. By promoting physical activity, healthy eating, adequate sleep, stress management, and mindful technology use, medical institutions can support the holistic well-being of students and cultivate a culture of self-care within the medical profession. Future research should focus on optimizing and addressing student participation in wellness programs implemented by institutions and designing programs that are not an additional burden to students.
